# Bad Effects of Animal Diet

**Published:** 1833

**Authors:** 


### II. Bad Effects of Animal Diet.

A Kentucky correspondent has sent us for publication, an article on the bad effects which result from so large a proportion of animal food, as is consumed by the people of the United States. It is a sensible production, on a subject of some importance, but does not present sufficient originality of thought or fact, to justify its insertion. The writer correctly observes, that "man is so great a slave to his appetites, that were a prophet to rise from the dead, and give warning of the bad effects of such indulgence, he would not be heeded." We concur entirely in the opinion, that one cause of the great prevalence and mortality of inflammatory diseases in the United States, is an excessive use of animal food, leading to plethora and a phlogistic diathesis. It should be regarded as a professional duty, on all suitable occasions, to warn our friends and patients against this abuse.

